# Oral SARS‐CoV‐2 reduction by local treatment: A plasma technology application?

**DOI:** 10.1002/ppap.202200196

**Published:** 2022-12-22

**Authors:** Thomas von Woedtke, Gülsah Gabriel, Ulrich E. Schaible, Sander Bekeschus

**Affiliations:** ^1^ ZIK Plasmatis, Leibniz Institute for Plasma Science and Technology (INP), a Member of the Leibniz Health Technologies Research Alliance Greifswald Germany; ^2^ Institute for Hygiene and Environmental Medicine Greifswald University Medical Center Greifswald Germany; ^3^ Department of Viral Zoonoses—One Health Leibniz Institute of Virology (LIV), A Member of the Leibniz Infections Research Alliance Hamburg Germany; ^4^ Institute of Virology University of Veterinary Medicine Hannover Hannover Germany; ^5^ Department of Cellular Microbiology Program Area Infections, Research Center Borstel, Leibniz Lung Center, A Member of the Leibniz Health Technologies and Leibniz Infections Research Alliances Parkallee Borstel Germany

**Keywords:** cold plasma, dielectric barrier discharges (DBD), plasma jet, plasma medicine, SARS‐CoV‐2

## Abstract

The SARS‐CoV‐2 pandemic reemphasized the importance of and need for efficient hygiene and disinfection measures. The coronavirus' efficient spread capitalizes on its airborne transmission routes via virus aerosol release from human oral and nasopharyngeal cavities. Besides the upper respiratory tract, efficient viral replication has been described in the epithelium of these two body cavities. To this end, the idea emerged to employ plasma technology to locally reduce mucosal viral loads as an additional measure to reduce patient infectivity. We here outline conceptual ideas of such treatment concepts within what is known in the antiviral actions of plasma treatment so far.

## Graphical abstract

Plasma could be a promising tool to locally reduce mucosal viral loads in the upper respiratory tract as an additional measure to reduce patient infectivity. Conceptual ideas based on direct plasma jet application to the mucosal tissue on the one hand or plasma‐treated aerosols or gases for inhalation purposes on the other are presented and discussed with respect to current knowledge on the antiviral effectivity of plasma treatment.

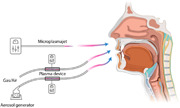

## 1 INTRODUCTION

Since March 2020, a pandemic of the coronavirus disease 2019 (COVID‐19) caused by the virus SARS‐CoV‐2 has dominated the world, resulting in several profound societal and scientific challenges. The virus is transmitted through aerosols and direct and indirect contact, as well as medical procedures and specimen handling. Therefore, distancing, wearing masks and gloves, handwashing and disinfection, and broad test strategies became the primary measures to prevent and embank virus spreading. However, from the beginning, there have been intensive efforts worldwide to investigate more specific, preventive, and curative measures to fight against SARS‐CoV‐2 and COVID‐19. Above all, comprehensive prevention by effective vaccination and causative treatment of the disease by newly developed antiviral drugs are the focus of medical research and pharmaceutical development.

**Figure 1:**
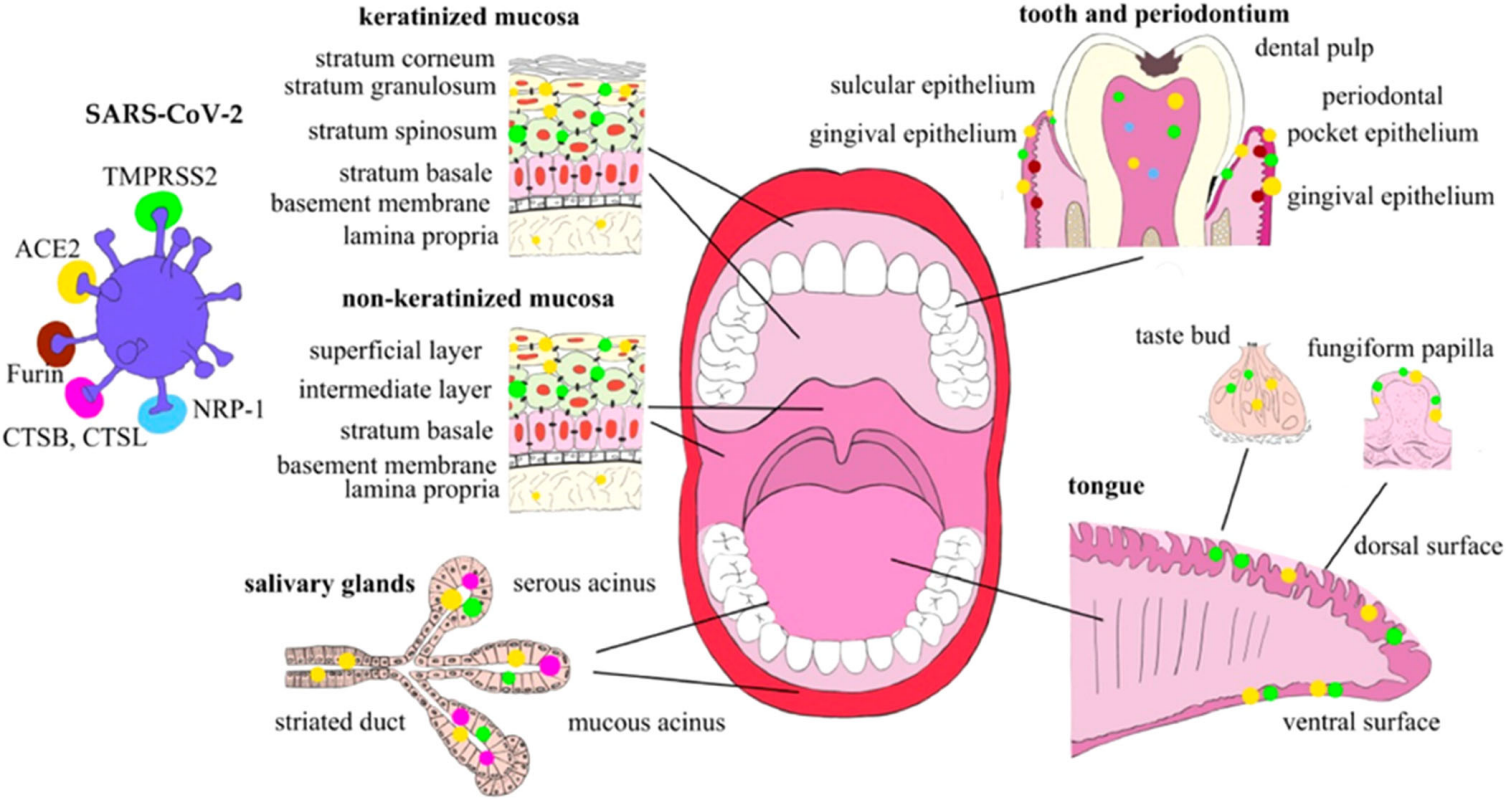
Expression of SARS‐CoV‐2 entry and transmission factors in the oral cavity structures. Reproduced from Drozdzik and Drozdzik^[^ ^2^ ^]^ licensed under a Creative Commons Attribution (CC BY 4.0).

It is well known that the nasal cavity, nasopharynx, oropharynx, and oral cavity play an important role in the entry and transmission of SARS‐CoV‐2 because of direct infection and replication in oral and nasal tissues.^[^ ^1^ ^]^ Important structures of the oral cavity and factors that are important for SARS‐CoV‐2 entry and transmission are summarized in Figure 1. Briefly, there are two viral entry pathways: receptor‐mediated endocytosis and membrane fusion mediated by a host‐cell‐derived transmembrane serine protease 2 (TMPRSS2).^[^ ^3^ ^]^ For both pathways, angiotensin‐converting enzyme 2 (ACE2) on the cell membrane plays a vital role as the sole binding partner of the spike protein (S) of the viral envelope.^[^ ^4^ ^]^ A host‐cell‐derived protease (i.e., an enzyme that catalyzes the hydrolytic breakdown of proteins), assumingly furin, cleaves S into the subunits S1 and S2. TMPRSS2 cleaves S2, thereby exposing the peptide and allowing host cell membrane fusion and invasion of the virus. Other tissue‐specific proteases (e.g., TMPRSS4 and TMPRSS11D) and endosomal proteases (e.g., CTSB, CTSL, and BSG) can also mediate virus entry. These SARS‐CoV‐2 entry factors were identified in the oral mucosa, tongue, salivary glands, and gingiva, pointing out the high susceptibility of this region to SARS‐CoV‐2 infection (Figure 1).^[^ ^2^ ^]^

Consequently, several local treatment measures for the primarily virus‐contaminated mouth and throat region are investigated.^[^ ^5^, ^6^ ^]^ Above all, a rationale for local antiviral treatment before oral dentistry procedures is discussed. Preprocedural antiseptic rinse is a well‐established method in dentistry to reduce microbial pathogens and, consequently, the risk of microbial transmission by salivary droplets and aerosols to medical professionals. Its application has also been suggested during the SARS‐CoV‐2 pandemic by a guideline of the German Society for Dental, Oral, and Maxillofacial Medicine (DGZMK, AWMF 083‐046) as well as the Center for Disease Control (CDC) and the American Dental Hygienists' Association (ADHA).^[^ ^7^, ^8^ ^]^ There are several in vitro studies on the antiviral effects of oral care products, antiseptic mouthwashes, and oral or nasal sprays.^[^ ^7^, ^8^, ^9^ ^]^ However, one systematic review of clinical evidence on oral antiseptics against coronavirus states sufficient in vitro evidence supporting the use of oral antiseptics like cetylpyridinium chloride (CPC), povidone‐iodine (PVP‐I), and hydrogen peroxide (H_2_O_2_) to reduce the viral load of coronaviruses, while in vivo efficacy and evidence is limited.^[^ ^10^ ^]^ Nevertheless, there is an anecdotal report on protecting inpatients and healthcare workers from COVID‐19 by daily use of hydrogen peroxide mouthwash and nasal rinse.^[^ ^11^, ^12^ ^]^ Yet, another report suggests only temporary inhibition of virus shedding of such an approach.^[^ ^13^ ^]^

## 2 ANTIVIRAL STRATEGIES AND COLD ATMOSPHERIC‐PRESSURE PLASMA (CAP)

In the early stages of discussion on measures against the pandemic, CAP was raised as a potential technology to reduce viral loads during COVID‐19 infections. CAP is a well‐studied effective antimicrobial tool. There are many publications on the potential of CAP to inactivate bacteria, including bacterial biofilms, fungi, and bacterial spores, and its potential practical application against those infections.^[^ ^14^, ^15^, ^16^ ^]^ Therefore, it was apparent to bring CAP into the discussion on promising strategies against COVID‐19.^[^ ^17^, ^18^, ^19^, ^20^ ^]^ A comprehensive review of the potential of CAP as part of strategies against viral infections with specific regard to SARS‐CoV‐2 was given previously.^[^ ^21^ ^]^ Briefly, antiviral plasma applications are suggested to prevent viral spreading by "classical" means of decontamination or disinfection of liquids, surfaces, or air and aerosol microdroplets. Hand disinfection by CAP‐based devices is also taken into consideration.^[^ ^17^, ^18^, ^21^ ^]^ Besides direct plasma application, also plasma‐treated (or "plasma‐activated") liquids are under discussion as potential antiviral disinfectants.^[^ ^22^, ^23^ ^]^ All these measures against viruses in the environment of infected and vulnerable people have to ensure the effective inactivation of viruses to block infectiousness by plasma treatment.

**Figure 2:**
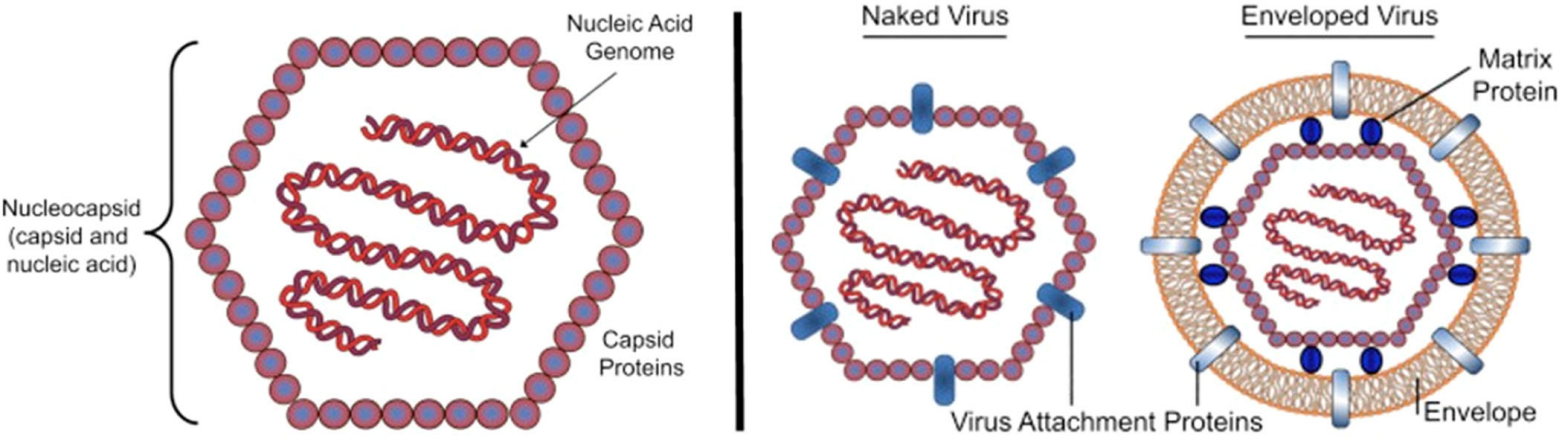
Basic virus architecture (left) and comparison between a naked and an enveloped virus particle (right). Reprinted from Louten^[^ ^29^ ^]^ with permission from Elsevier.

Several in vitro studies suggest the antiviral effects of CAP, as summarized before.^[^ ^21^, ^24^, ^25^ ^]^ Similar to the inactivation of microorganisms, antiviral CAP effects likely depend on the plasma device geometry, plasma and electric parameters, type of virus, treatment conditions, and matrix (virus environment). Experimental work with viruses pathogenic in humans requires specialized laboratory equipment to reduce health risks to research staff. Therefore, research on antiviral CAP effects is often done with surrogate viruses like animal viruses or bacteriophages.^[^ ^26^, ^27^ ^]^ According to the present knowledge, the inactivation of microorganisms is based on the plasma‐induced impact on bacterial structures (e.g., permeabilization or damage of cell membrane or wall, modification or damage of intracellular proteins, impact on DNA), leading to a fatal impact on microorganism's cell integrity and metabolism.^[^ ^28^ ^]^ In contrast, viruses do not actively metabolize and depend on host cells for replication. In naked (nonenveloped) viruses, a proteinaceous capsid is the outermost layer of the virus enclosing the genetic material (RNA or DNA). An additional phospholipid and glycoprotein‐containing membrane derived from the host cell surrounds enveloped viruses. Both the capsid and the envelope are mostly responsible for the host cell specificity of the virus and its ability to attach to corresponding host cell receptors to facilitate its entry into the host cell via virus attachment proteins (Figure 2; see Louten^[^ ^29^ ^]^ for details). Consequently, any measures to inactivate viruses have to be based on the structure and integrity of the virus' capsid or envelope to prevent its attachment to host cells or the integrity of the genetic material to make virus replication impossible. As well as in the case of antimicrobial CAP effects, antiviral plasma effects are thought to be mediated via plasma‐generated reactive oxygen and nitrogen species (RONS) affecting proteins and deficiently‐protected nucleic acids.^[^ ^21^, ^25^, ^26^, ^27^ ^]^ In SARS‐CoV‐2, an RNA virus, the so‐called spike protein is the key target for the human receptor ACE2, mediating membrane fusion and viral entry into the host cell.^[^ ^5^ ^]^ There are first reports on the potential modification of this spike protein by treatment with CAP directly or by plasma‐treated liquids to interfere with viral entry,^[^ ^23^, ^30^, ^31^ ^]^ albeit exact molecular models and mechanisms are still awaited. Furthermore, a recent study demonstrated that direct CAP exposure and treatment of cells by plasma‐treated cell culture medium reduced the number of cells becoming SARS‐CoV‐2 infected, and—presumably oxidative stress‐mediated—ACE2 internalization was proposed as a mechanism of action.^[^ ^32^ ^]^

## 3 MOUTH AND THROAT AS TARGET REGIONS FOR CAP TREATMENT

Generally, it is understood that cell culture settings to investigate plasma action on virus‐infected cell lines in vitro do not fully recapitulate the full arsenal of plasma components effective during the treatment of tissue or model tissues.^[^ ^33^ ^]^ Localized CAP application to the patients' skin in the clinical setting was beneficial in the case of herpes simplex,^[^ ^34^ ^]^ herpes zoster,^[^ ^35^ ^]^ and warts,^[^ ^36^, ^37^ ^]^ suggesting that CAP may also be effective against virus‐infected cells in diseased tissue or may be able to inactivate extracellular viral particles. With regard to the plasma‐mediated inactivation of viruses on the oral mucosa, there are no studies available so far. Several points are to be discussed in such a regard. First, as outlined above, it remains to be established what the detailed mechanisms of such antiviral activity are. This can be only speculated about and will not be detailed any further here. The second question is, who would be the patient group targeted by such treatment? Severely ill SARS‐CoV‐2 patients display deeply manifested infections in the lower respiratory tract and show lung tissue destruction due to exacerbating inflammatory host responses. Considering the many drugs and medical devices already operated in intensive care units, and given the potential of RONS to exacerbate local inflammation in the lung, it might be conceivable that patients with relatively mild symptoms dominated by upper respiratory tract infections might benefit from additional therapeutic options, for instance, to reduce their infectivity in delicate environments (e.g., retirement homes). Therefore, the third question is how plasma technology may be useful in such an application outside intensive care units.

Several types of plasma sources exist for biomedical applications, with the main types—in simple words—being dielectric barrier discharges (DBDs)^[^ ^38^ ^]^ and plasma jets.^[^ ^39^ ^]^ Plasma jets show excellent pen‐like handling to precisely reach and expose the target tissue to plasma. In addition, jet plasmas have a superior ability to penetrate small cavities and reach uneven surfaces. Hence, it seems conceivable that the, for example, oral mucosa receives direct jet plasma treatment, ideally with miniaturized plasma jets, as recently engineered.^[^ ^40^ ^]^ However, from a practical perspective, a complete plasma exposure of the entire mucosal surface may be limited for two reasons. First, the surface area of the nasopharyngeal mucosa is about 50 cm^2^,^[^ ^41^ ^]^ translating to a reasonably long 25 min plasma treatment time (supposing 30 s plasma treatment per cm^2^). Second, the nasopharyngeal cavity has complex anatomical shapes, which likely will make a uniform plasma treatment across most surfaces difficult to achieve. Third, the nasal cavities and their mucosa are partially too small, even for smaller plasma devices to enter. Having said that, and considering the flat and rather stiff geometry of DBDs, direct treatment of the, for example, oral mucosa by such plasma sources in patients seems even more unlikely.

Notwithstanding, it should be mentioned that direct plasma application of the human mucosa is safe. Several studies have underlined this notion.^[^ ^42^, ^43^, ^44^, ^45^, ^46^, ^47^ ^]^ For instance, the argon gas‐driven cold atmospheric plasma jet kINPen and a helium and nitrogen‐driven microwave plasma source were tested on the buccal cheek mucosa in mice for its safe application. It was found that plasma application temporarily led to mild superficial mucosal damage immediately after treatment that healed completely within 1 week.^[^ ^42^ ^]^ Applying these plasma devices to oral mucosae in mice repeatedly on a monthly frequency for 1 year, all mice tolerated the treatment well, and none of the animals showed any signs of carcinogenic effects.^[^ ^43^ ^]^ Another study using the surface microdischarge MiniFlatPlaSter, a type of DBD, driven in atmospheric air to treat human oropharyngeal mucosa ex vivo for up to 60 s showed minor adverse effects on mucosal cells only.^[^ ^44^ ^]^ A similar study performed with the approved medical product kINPen MED on healthy mucosal tissue of the maxillo‐facial region showed that 30 s of ex vivo plasma treatment did not increase the number of apoptotic cells within the exposed region in a significant manner. Similarly, plasma treatment did not increase the levels of released cytochrome c, indicative of necrosis. The same study also showed that the production of neither pro‐inflammatory tumor necrosis factor‐alpha nor interferon‐gamma nor anti‐inflammatory interleukin 10 was significantly changed between untreated and plasma‐treated tissue,^[^ ^45^ ^]^ indicating a lack of severe local reactions in response to multi‐ROS exposure. Likewise, another report using the kINPen found no histological alterations in plasma‐treated oral mucosa exposed ex vivo.^[^ ^46^ ^]^ The same report did suggest keratin 14 expressions are altered upon plasma treatment, while the levels of the DNA double‐strand break indicator gamma H2A.X were unaffected by plasma treatment, suggesting its safe application and distinct matrix‐remodeling responses. In ex vivo kINPen plasma‐treated healthy human oral mucosa, it was moreover found that the distribution of T‐cell subtypes, mainly T‐helper and cytotoxic T‐cells of memory and naïve phenotypes, were unchanged.^[^ ^47^ ^]^ This was a surprise as we had previously identified primary T‐cells as the most sensitive human cell type in terms of showing cytotoxic effects to in vitro plasma treatment. It is evident that these few investigations do not allow a general estimation of the compatibility of CAP application on the mucosa of the mouth and throat region but show that such an application should be quite feasible.

**Figure 3:**
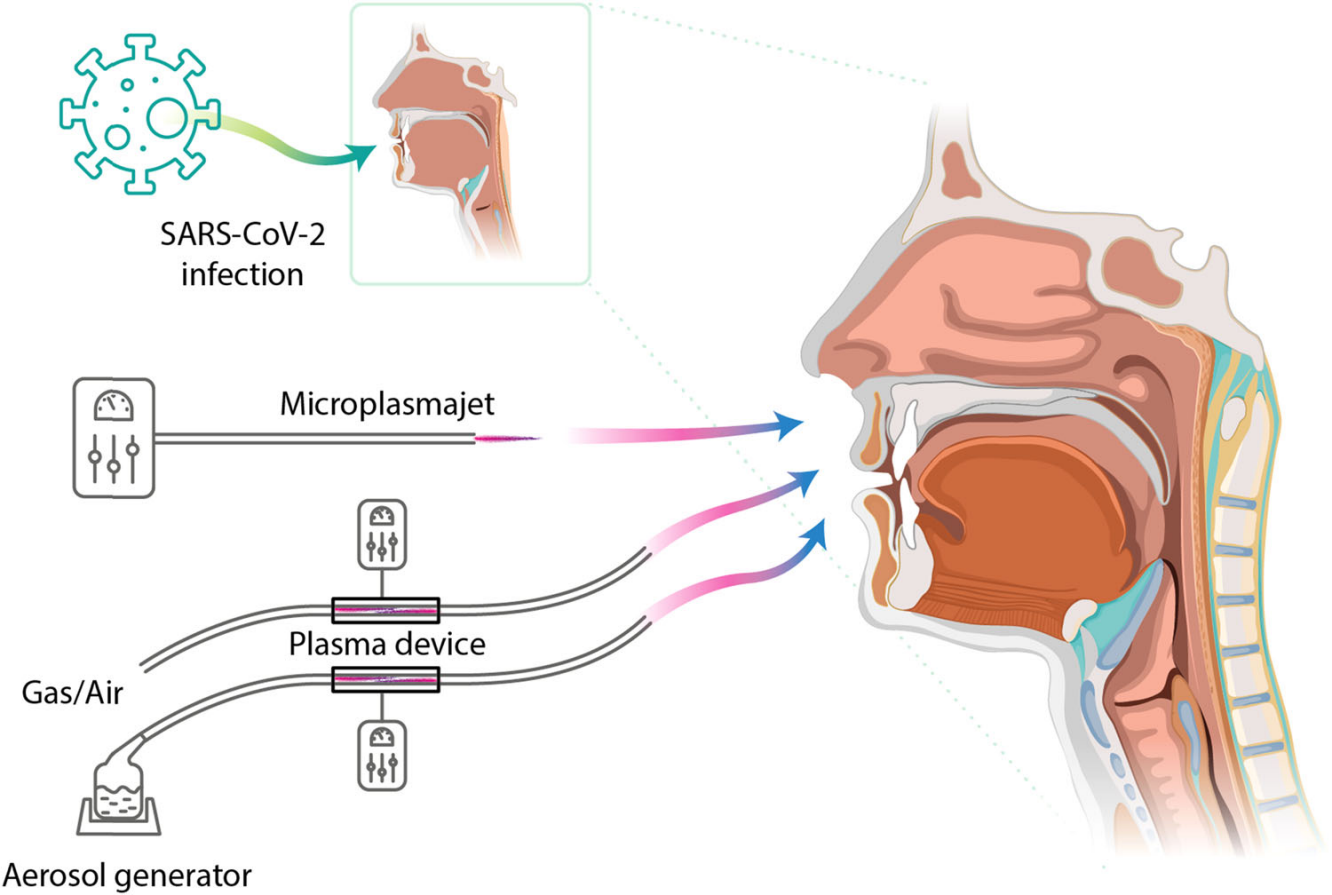
Possible plasma application modes to the SARS‐CoV‐2‐infected oral and nasopharynx mucosa for local viral load reduction via microplasma jet applied directly to the tissue or plasma‐treated aerosols or gases for inhalation purposes.

In contrast to a hypothetic direct plasma treatment of SARS‐CoV‐2‐infected human oral and nasopharyngeal mucosa, another therapeutic approach would be the generation of plasma‐treated air or aerosol (Figure 3). This would be beneficial because direct contact of plasma with the target does not necessarily avoid the mentioned problem of long plasma treatment times to cover the complete mucosa. Using an external plasma device, for example, a flow‐driven DBD,^[^ ^48^ ^]^ air or an aerosol, that is, liquid droplets carried by an airflow will be plasma treated and afterward applied to the mouth and throat region via a suitable application system. The concept is to "flood" the complete oral and nasopharynx to achieve an effect on the entire mucosa.

Plasma‐treated air and plasma‐treated liquids are well‐known antimicrobial effective agents as demonstrated for several applications, for example, in food decontamination.^[^ ^49^, ^50^ ^]^ However, medical applications of plasma‐treated air have rarely been described. Yet, a German company reports on its website (https://www.terraplasma-medical.com/intensive-medicine/) on a plasma‐treated air‐based system under investigation for intensive care application in mechanical ventilation, but valid proof‐of‐concept studies towards efficacy and safety are still awaited. Plasma‐treated liquids are under investigation mainly for cancer treatment as potential therapeutic alternatives to treat tumors located in body regions that are difficult to access by direct treatment like plasma application or surgery.^[^ ^51^, ^52^, ^53^ ^]^ However, it will be a scientific and technological challenge to transfer these experimental and practical experiences on the biological effects of plasma‐treated liquids to aerosol‐based approaches.^[^ ^18^, ^54^, ^55^, ^56^, ^57^ ^]^

## 4 CURRENT CHALLENGES

As a general fact, it has to be emphasized that all biological plasma effects are strongly dependent on basic parameters like plasma device configuration, plasma parameters, working gas, treatment time, and, resulting from this, the composition of the reactive effectors generated. Therefore, the technological development of plasma devices adapted to this specific application must be accompanied by a comprehensive characterization of their specific plasma physical properties and achieved biological effects. Analogy conclusions based on individual studies with specific plasma sources to other plasma devices must be treated with great caution.

If such devices are dedicated to reducing the SARS‐CoV‐2 load in the upper respiratory tract, specific efficacy against this virus must be demonstrated. Here, too, analogy conclusions from studies with model viruses can only have an orienting character. In this context, proof of antiviral plasma efficacy must not just be realized by simple in vitro studies, for example, using virus suspensions, but must be demonstrated in suitable experimental infection models in vitro and in vivo. On the one hand, coculture setups based on air–liquid interface in vitro models would be suitable for this.^[^ ^58^ ^]^ However, the actual preclinical proof of efficacy in vivo has to be achieved in a suitable animal model. As small animal models, ferrets and hamsters can be used for experimental SARS‐CoV‐2 infection studies.^[^ ^59^ ^]^ Such animal models are indispensable to prove not only the local reduction of virus load in the mouth and throat region but also to demonstrate any efficacy to prevent or at least alleviate viral disease. Additionally, employing such infection models is the only way to determine the sustainability of plasma treatment, that is, the necessity of repeated applications to reduce the virus burden and prevent further development of the virus disease.

Several reports have described vesicular bullous lesions and ulcerations as concomitant effects of oral manifestations of SARS‐CoV‐2 infections.^[^ ^2^, ^60^ ^]^ Therefore, in addition to the local antiviral efficacy of CAP, its anti‐inflammatory and tissue regenerating, that is, healing, effects should be considered. This is the most important and unique CAP effect in wound healing.^[^ ^61^ ^]^ On the other hand, as demonstrated in the first experimental plasma applications, slight mucosal injuries can occur as temporary side effects. Consequently, any aspects of mucosal compatibility have to be taken into account.

Finally, aspects of plasma compatibility apply not only to the mucous membrane of the mouth and throat but also to the respiratory toxicity of plasma‐generated gas species such as ozone or nitrogen oxides. Even if a local plasma application in the mouth and throat region is intended, an inhalation of plasma‐generated gas species cannot be completely excluded. In the animal studies mentioned above,^[^ ^42^, ^43^ ^]^ no weight loss in plasma‐treated mice was detected. This is the first indicator that this treatment did not influence the general well‐being of the animals. The quality and quantity of toxic gas species generation depend on plasma operation characteristics and the working gas used for plasma generation. Nevertheless, the investigations here should focus primarily on ozone (O_3_) and nitrogen oxides (NO*x*). In general, ozone is a powerful oxidant causing respiratory toxicity. However, most studies are related to ozone as an environmental pollutant and the consequences of any long‐term exposure. Consequently, health‐protecting threshold levels of the environmental impact of ozone are in the range of 0.06 ppm as an 8‐h mean concentration. There are few studies on short‐term, acute ozone effects that may be considered differently due to effective antioxidant mechanisms in the lung^[^ ^62^, ^63^, ^64^ ^]^ (https://www.ncbi.nlm.nih.gov/books/NBK430751/). The situation is similar to nitrogen oxides. Threshold values of about 30–60 ppb refer to outdoor ambient concentrations^[^ ^65^ ^]^ (https://www.ncbi.nlm.nih.gov/books/NBK554539/). Consequently, the respiratory toxicity of plasma‐generated gas species has to be investigated in detail related to the specific conditions of use to guarantee a safe plasma application, also concerning lung compatibility on the one hand. On the other hand, such studies could also be valuable to estimate if such plasma treatment might even be beneficial to limit virus loads in the lower respiratory tract. This is true above all for the approach based on plasma‐treated air or aerosol.

## 5 CONCLUSIONS AND OUTLOOK

A global pandemic, like COVID‐19, needs a broad approach of measures to protect and fight against its consequences for human health and well‐being. CAP could be one tool to contribute to an effective defense strategy. Besides several possibilities to use CAP for antiviral disinfection and decontamination, a preventive or therapeutic plasma application in the upper respiratory tract should also be considered. Both, its antiviral activity and its emerging compatibility with mucosa support this approach. However, to come to an effective and applicable practical solution, several challenges in preclinical and clinical research, as well as in plasma technology, have to be faced.

In general, respiratory tract infections are highly contagious due to their ability to transmit via aerosols. Therefore, local anti‐infective treatments will be useful in the future, far beyond COVID‐19. Consequently, research and development in this field should be strengthened with cold atmospheric plasma application to the oral and respiratory tract as a promising anti‐infective tool.

## CONFLICT OF INTEREST

The authors declare no conflict of interest.

## Data Availability

Data sharing are not applicable to this article as no datasets were generated or analyzed during the current study.
